# Empirical Investigations during WEDM of Ni-27Cu-3.15Al-2Fe-1.5Mn Based Superalloy for High Temperature Corrosion Resistance Applications

**DOI:** 10.3390/ma13163470

**Published:** 2020-08-06

**Authors:** Vivek Aggarwal, Catalin Iulian Pruncu, Jujhar Singh, Shubham Sharma, Danil Yurievich Pimenov

**Affiliations:** 1 Department of Mech. Engg., IKG Punjab Technical University, Jalandhar-Kapurthala Road, Kapurthala 144603, India; agarwalz_v@yahoo.com (V.A.); jujharsingh2085@gmail.com (J.S.); shubham543sharma@gmail.com (S.S.); 2 Mechanical Engineering, Imperial College London, Exhibition Rd., London SW7 2AZ, UK; 3 Mechanical Engineering, School of Engineering, University of Birmingham, Birmingham B15 2TT, UK; 4 Department of Automated Mechanical Engineering, South Ural State University, Lenin Prosp. 76, Chelyabinsk 454080, Russia; danil_u@rambler.ru

**Keywords:** Monel K-500, wire electric discharge machining (WEDM), response surface methodology (RSM), central composite design (CCD), surface roughness

## Abstract

Monel K-500, a nickel–copper based alloy, is a very hard and tough material. Machining of such hard and tough materials always becomes a challenge for industry and this has been resolved by wire electric discharge machining (WEDM), a popular non-conventional machining method used for machining tough and hard materials having complex shapes. For the first time reported in this present research work is an experimental investigation executed on Ni-27Cu-3.15Al-2Fe-1.5Mn based superalloy using WEDM to model cutting rate (CR) and surface roughness (SR) using response surface methodology (RSM). The process parameters have been selected as pulse-on time, pulse-off time, spark-gap voltage and wire-feed rate. Experiments have been planned according to the central composite design (CCD). The results show that pulse-on time has a direct effect on CR while the pulse-off time has a reverse effect. The CR increases as pulse-on time increases, and decreases as pulse-off time increases. SR increases as pulse-on time increases, and decreases as pulse-off time increases. Furthermore, increase in spark-gap voltage decreases CR and SR both. The wire feed-rate has a negligible effect for both the response parameters. The optimized values of CR and SR achieved through multi-response optimization are 2.48 mm/min and 2.12 µm, respectively.

## 1. Introduction

Jet engines, gas turbines and rocket applications require materials that possess high corrosion, creep, oxidation resistance and fatigue strength at temperatures above 1100 °C. One category of materials which provide such properties is the superalloys. A superalloy has a very high strength as well as creep resistance at raised temperatures. The capability of superalloys to keep their mechanical properties at raised temperatures hinders their machinability. Hence, they are also named as hard-to-cut alloys [1,2]. Superalloys are categorized into iron, cobalt, and nickel-based alloys. The nickel-based alloys are much tougher and stronger in contrast to iron and cobalt-based alloys due to their outstanding mechanical properties, and resistance to creep particularly at elevated temperatures. Attributable to these reasons, nickel-based superalloys are of high importance and commonly used in industry [3]. Among nickel-based superalloys, Monel K-500 is a well-known nickel-chromium superalloy with additions of aluminum and titanium which make it strong, hard, excellent corrosion resistant, resistant to sour gas environments and stress corrosion cracking. This material is widely used in marine environments due to its excellent corrosion resistance, and is often utilized in high-velocity seawater and in stagnant or slow-moving seawater. Furthermore, it shows excellent strength and toughness levels, good resistance to non-oxidising acids, alkali and salts and a high retention of properties at elevated temperatures [4]. Therefore, all these properties make it an appealing material for applications, such as, pump shafts, springs, valves and impellers, marine propeller shafts, oil well tools and electronic components. Machining of Monel K-500 is constantly an area of active research attributable to demand of this material because of its various industrial applications. The machining techniques are separated into two divisions which incorporate conventional and non-conventional machining. Conventional machining has issues like shorter tool life, excess material wastage, poor surface finish, low productivity and excessive tool wear [5,6] leading to the evolution of non-traditional machining which involves no physical contact amongst the workpiece and tool. These processes provide us complex contours with higher-dimensional precision and surface finish in high-strength materials. Wire electric discharge machining (WEDM) is one such extensively used non-conventional machining technique which uses spark erosion process to cut complicated profiles in hard, tough and conductive metals [7]. Cutting of superalloys, ceramics and composite are widely chosen using this process [8,9]. During machining with WEDM, a wire electrode of about 0.05–0.3 mm in diameter is utilized to process a work piece, which is placed on self-controlled work table actuated by CNC Technology. To extract the debris from the machining area, the dielectric liquid (mostly de-ionized water) is continuously discharged [10,11]. The channel of plasma is produced between the workpiece and wire which raise the temperature up to 12,000 °C, leading to erosion of the material [12]. Attributable to innumerable input parameters and random behaviour of the WEDM process, even a best in class WEDM operator is rarely capable of accomplishing the optimum execution [13]. A successful strategy to deal with this issue is to decide the relationship of the output and input measures utilizing an appropriate modelling strategy.

In this section, the research activities made by various authors for machining of steel, ceramic, composites, tungsten, and few superalloys with WEDM have been presented. Kuriakose et al. [14] modelled a process of WEDM by applying the Taguchi method to examine the impact of pulse-on time (Ton), pulse-off time (Toff), and WT (Wire Tension) upon the cutting rate (CR) and surface roughness (SR) of Ti-6Al-4V alloy. Sarkar et al. [15] expanded mathematical equations using additive model to forecast the cutting speed, and dimensional variation in terms of Ton, Toff, peak current (IP), and dielectric flow rate while WEDM of γ-titanium aluminide material. Ramakrishnan and Karunamoorthy [16] established ANN (Artificial Neural Network) models to forecast the effect of Toff, Ton, and IP on SR and MRR (Material Removal Rate) by WEDM of Inconel 718. Kondayya and Krishna [17] applied genetic the algorithm-II for modeling the MRR and SR during WEDM processing of AISI D3 steel. The Ton, Toff, WT and WF (Wire Feed Rate) were used as input factors. Shahali et al. [18] used Taguchi technique and micro-genetic algorithm for analyzing the impact of power, voltage and Toff on SR and white layer thickness during WEDM of DIN 1.4542 stainless-steel. Prasad et al. [19] also used Taguchi technique to experimentally find the impact of Toff, Ton, IP and servo voltage (SV) on MRR and SR during WEDM of Ti-6Al-4V. Mandal et al. [20] applied a central composite rotatable design of response surface methodology (RSM) during cutting of Nimonic C-263 with WEDM for investigating the effect of Ton, Toff, SV and flow rate of dielectric on CR, SR, and wire wear ratio. Tonday and Tigga [21] used WEDM for experimental work on Inconel 825 and employed Taguchi and analysis of variance (ANOVA) for exploring the influence of Ton, WT, SV and flushing pressure of dielectric upon MRR and SR. Rao and Venkaiah [22] applied a central composite design of RSM to investigate parametric impact of Toff, Ton, IP and SV on micro-hardness, and SR during WEDM of Inconel 690. Soni et al. [23], Muni et al. [24] and Gill et al. [25] attempted to explore the impact of SV and Ton on the CR and SR of Ti50Ni49Co1 shape memory alloy during processing with WEDM.

To summarize, most of the research efforts have been carried out on WEDM of steel, ceramics, composites, tungsten, titanium and few superalloys namely Ti-6Al-4V alloy, γ-titanium aluminide, Inconel 718, AISI D3 steel, DIN 1.4542 stainless-steel, Nimonic C-263, Inconel 825, Inconel 690, Ti50Ni49Co1 shape memory alloy etc. Furthermore, in these research efforts, effect of numerous process parameters on output parameters of WEDM have been explored. To the extent that Ni-27Cu-3.15Al-2Fe-1.5Mn based Monel K-500 superalloy is concerned, relatively less research has been undertaken in spite of its huge application area. Further, as far as authors knowledge is concerned, little or no research has been reported on the empirical modeling of CR and SR during WEDM of Ni-27Cu-3.15Al-2Fe-1.5Mn based superalloy. Additionally, at present, there is no suitable model for this process because of numerous factors and stochastic process which also demands to perform research work on experimental studies of WEDM. Based on the recognized research gap, accordingly, the aim of this study is to perform WEDM on Ni-27Cu-3.15Al-2Fe-1.5Mn based superalloy so as to create exact models for CR and SR to research the impact of the various input parameters viz. Ton, Toff, SV and WF on these output measures. Further, multi-objective optimization has been executed using multi-response approach known as desirability function.

## 2. Materials and Methods

The procedure employed for the conduct of experiment is detailed below.

### 2.1. Experimental Setup

The experimental setup consists of following components:

- Wire electrical discharge machine
- Work material
- Tool
- Dielectric fluid

#### 2.1.1. Wire Electrical Discharge Machine

**Figure 1:**
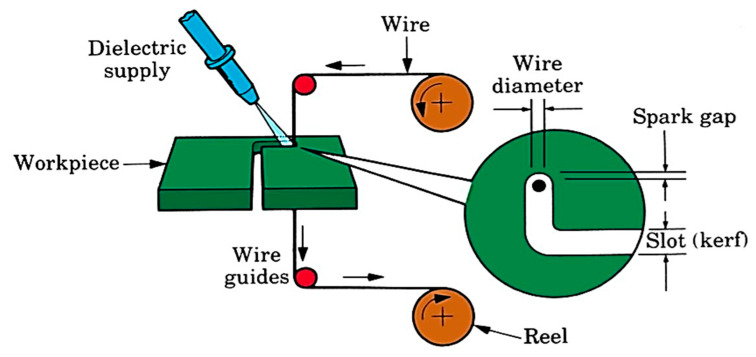
Wire electric discharge machining (WEDM) schematic diagram.

**Figure 2:**
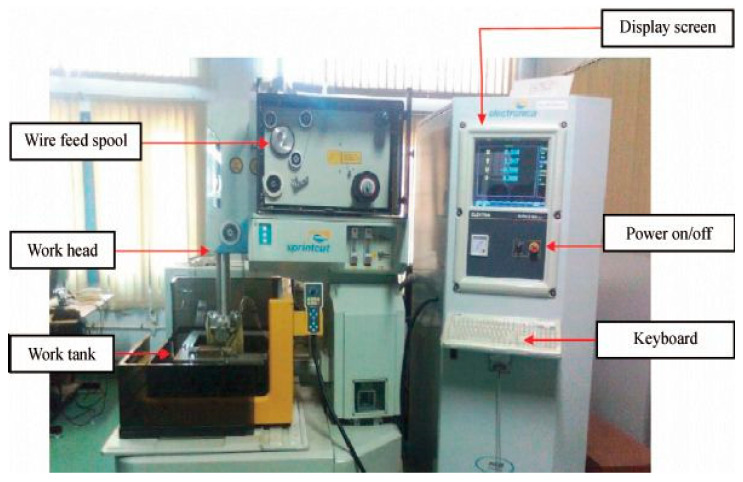
WEDM (SPRINTCUT-734).

**Figure 3:**
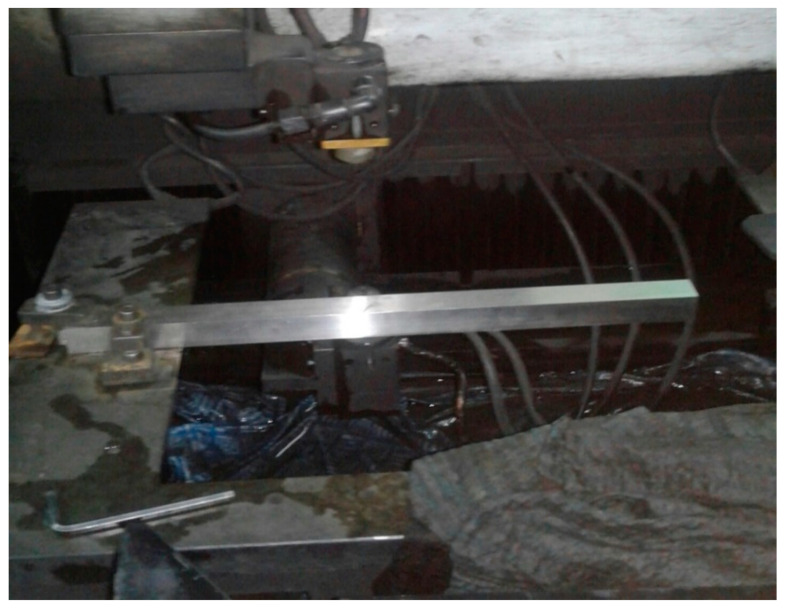
Experiment set up on WEDM.

For WEDM, Sprintcut 734 is utilized to execute experimentation. The machine consists of a power supply, dielectric supply and wire feed units. The schematic arrangement of this process is indicated in Figure 1. The proper arrangement has been made in this machine to guide the wire for machining the required profile on the work which is fixed on the work table with clamps. The servo motor rotates according to the signal of microcontroller and, furthermore, this motion is transmitted to the work table for executing the machining operation. Experimental work being done on WEDM machine is shown in Figure 2 and Figure 3.

#### 2.1.2. Work Material

In the current investigation, the work piece selected for the experimental work is a single piece rod of Monel K-500 having size 24 mm × 24 mm × 300 mm and its chemical composition, which has been tested utilizing the optical emission spectrometer is enlisted in Table 1. All the experimental work on wired electric discharge (SPRINTCUT-734) system was carried out.

#### 2.1.3. Tool

For this experimental work, zinc-coated 0.25 mm brass wire was used as the wire electrode.

#### 2.1.4. Dielectric

In the WEDM, deionized water is taken as a dielectric. It provides cooling to the workpiece and tool and also takes away the removed material from the cutting area. The thermal conductivity of dielectric fluid is 20 mho.

### 2.2. Selected Input Parameters

#### 2.2.1. Pulse-On Time

Pulse-on time (Ton) is the duration of time for which the current is flowing in each cycle. For the present experimentation, five different values of pulse-on time were selected out of 10 values available on the WEDM by conducting preliminary trails. Machining of the test specimen was performed by changing pulse-on time while maintaining others parameters constant at the middle level. It was noticed that at pulse-on time of less than ‘108 µs’ and more than ‘124 µs’ the machine does not work efficiently. So, five values varying from 108–124 µs for pulse-on time were selected.

#### 2.2.2. Pulse-Off Time

Pulse-off time (Toff) is the time interval between two simultaneous sparks. So, the maximum value of pulse-off time was 58 µs at which WEDM cutting process can be performed without any loss in performance. The low and high pulse-off time values were 42 µs and 58 µs respectively and other values were selected in the same way as done for pulse-on time. So, the five different values of pulse-off time calculated on the basis of averaging were 42, 46, 50, 54 and 58 µs.

#### 2.2.3. Spark Gap Voltage

Spark gap voltage (SV) indicates the theoretical voltage difference between wire electrode and workpiece during erosion. The low and high values of voltage selected during preliminary experimentation were 24 volts and 72 volts due to their efficient machining. The other values of voltage were calculated to be equal to 36 volts, 48 volts and 60 volts as per details as follows. The average of minimum (1st) level and maximum (5th) level is the 3rd level (i.e., 48 volts) and the average of minimum (1st) level and middle (3rd) level is the 2nd level (i.e., 36 volts). In the same way, the average of the 3rd level and 5th level is the 4th level (i.e., 60 volts).

#### 2.2.4. Wire Feed Rate

Wire feed rate (WF) is rate at which wire moves through the wire guides and is fed continuously for sparking. In this process, wire advances on roller towards target for generating the required discharge for spark erosion. The wire is feed on WEDM at the rate of 12 m/min. The least and largest value of wire feed selected were 4 m/min and 12 m/min. The other values of wire feed were calculated as 6, 8 and 10 m/min as per details as follows. The average of minimum (1st) level and maximum (5th) level is the 3rd level (i.e., 8 m/min) and the average of minimum (1st) level and middle (3rd) level is the 2nd level (i.e., 6 m/min). In the same way, the average of the 3rd level and 5th level is the 4th level (i.e., 10 m/min). Table 2 lists input parameters along with their working range.

### 2.3. Experiment Plan

Current investigations have been conducted by considering parameters such as Ton, Toff, SV and WF as input variables and its impact on the productive variables like CR and SR. The modeling equations have been developed as per the application of the RSM technique of optimization. The coded and real values of input variable at different levels as suggested by response surface methodology are given in Table 2. Fixed parameters values are presented in Table 3. Thirty trials were executed as per the RSM approach and the design matrix is exhibited in Table 4. The randomization of experimental run was executed to eliminate biasing. The specimen was cleaned and grinded to flatten the surface to make sure that its surface is free from foreign particles. For each specified combination, same tool is used to keep experimental conditions same. The CR and SR were measured at each specified region.

## 3. Results and Discussion

The CR and SR have been calculated for each of 30 experiments and are shown in Table 4. With reference to experiment data, mathematical analysis has been performed using the software Design Expert (DX-8061). The regression equation for CR and SR has been obtained in term of input parameters. ANOVA has been executed to establish the significance of input variables and the confidence level of their effect. Statistical inference has been drawn in respect of model adequacy, precision, lack of fit etc. The impacts of input variables on response characteristics has been discussed with response surface graphs.

### 3.1. Statistical Observations for Cutting Rate (CR)

The model F value of 128.04 with its Prob > F value less than 0.0001 as exhibited in Table 5 indicates that the model is significant for cutting rate as it demonstrates that the terms in the model have a significant effect on the response. There is just a 0.01% possibility that “Model F-Value” of this large may happen due to noise. Furthermore, the model F value is calculated as ‘model’ mean square divided by ‘residual’ mean square. Similarly, an F value on any individual factor term is calculated as the term mean square divided by the residual mean square. The F value test compares the model (or term) variance with the residual variance. If the variances are nearly same, the ratio will be close to one and it is less likely that the model (or any of factor terms) has a significant effect on the response. A particular source of variation may be significant if the calculated F value at a certain confidence level is greater than the tabulated F value at the same confidence level. Confidence level is chosen to be 95 % in this study. If Prob > F value of the model is considerably less than 0.05 (i.e., at the 95% confidence level), then the terms in the model have a significant effect on the response [26,27]. The “Prob > F” under 0.0500 show model entities are important. For this situation A, B, C, D, AB, AC, BC and A^2^ are noteworthy model items. Value more than 0.1000 showed that these entities are not important. There is very less severity of importance of these terms. The “Lack of Fit F-esteem” of 2.52 infers that it is not noteworthily comparative to pure error. Non noteworthy lack of fit is acceptable as we need the model to fit.

The 0.9503 Predicted R^2^ is fairly in line with the 0.9723 Revised R^2^; i.e., this difference is less than 0.2. Adeq-Precision tests the ratio of a signal-to-noise. It is important to get a ratio greater than 4. The 41.642 ratio points to an adequate-signal. The model can be used to traverse space in design.

### 3.2. Regression Equation for CR

The response equation for CR in terms of input variables is given as under:

CR = 4.73455 − 0.344332 × Ton + 0.444427 × Toff + 0.057960 × SV + 0.020625 × WF − 0.004883 × Ton × Toff − 0.001107 × Ton × SV + 0.000924 × Toff × SV + 0.003225 × Ton^2^

**Figure 4:**
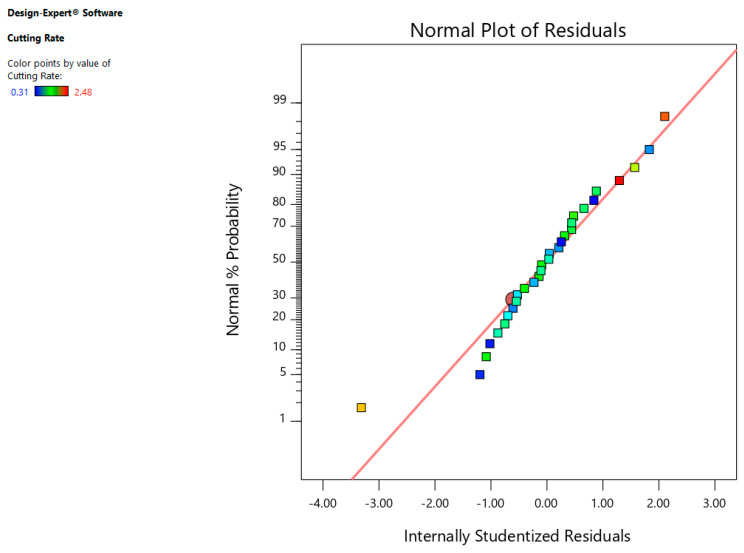
Normal chart of residuals for CR.

**Figure 5:**
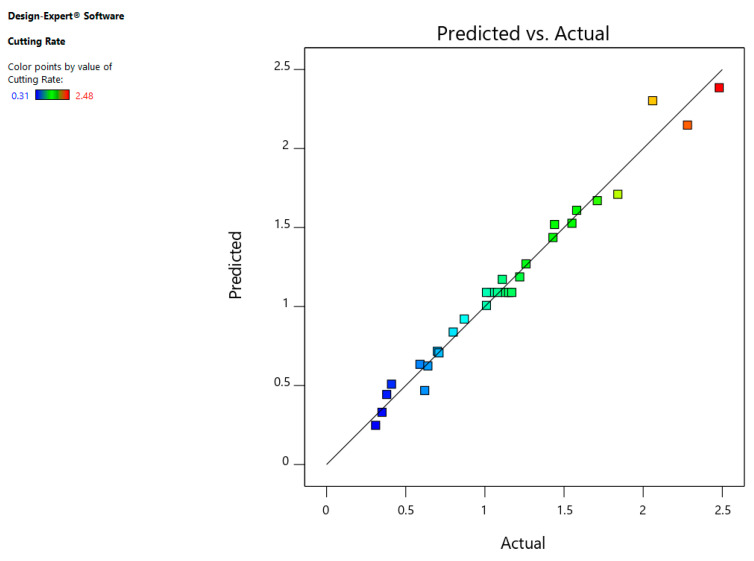
Predicted versus actual plot for CR.

The normal probability residual plot indicates that errors are normally scattered as shown in Figure 4. Furthermore, Figure 5 indicates that that whole experimental values and determined values from the equation are within close range which is the sign of better correlation among the aforementioned values.

### 3.3. Interaction Influence of Process Variables on CR

**Figure 6:**
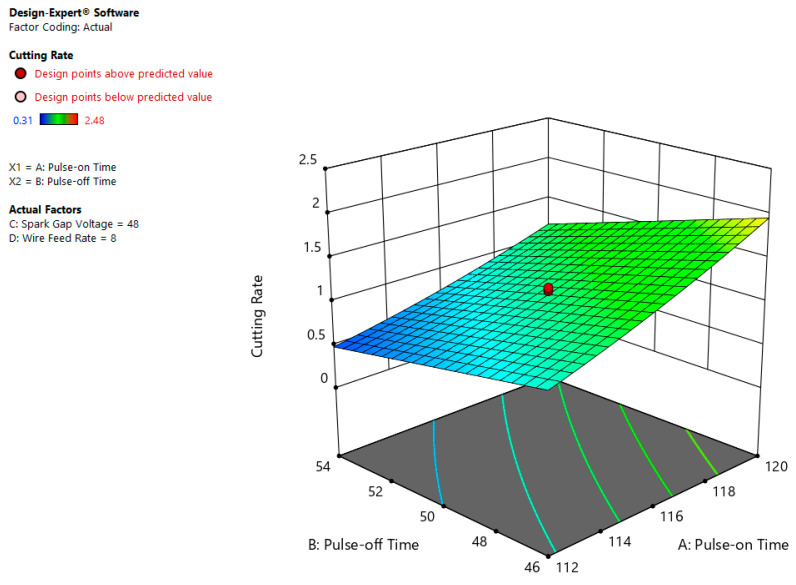
Interactive effect of pulse-on time (Ton) and pulse-off time (Toff) on CR.

The collaboration impact of Ton and Toff on CR (Figure 6) shows that CR goes to most highest value of about 2.0 mm/min at a large estimation of Ton (120 µs) and less estimation of Toff (46 µs), however CR value is at least level, when Ton is lowest (112 µs) and Toff is highest (54 µs). This is because of the reality that the low Ton and high Toff leads to erosion for a short time [8].

**Figure 7:**
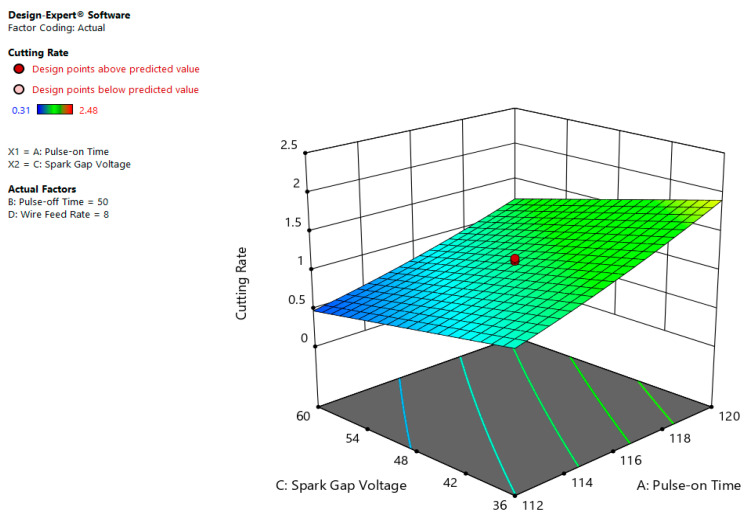
Interactive impact of Ton and SV on CR.

The correlational chart shown in Figure 7 indicates that maximum CR of about 2.0 mm/min occurs at large value of Ton (120 µs) and minimum magnitude of SV (36 volt). The lower SV value means the least gap between work and tool. As the gap is minimum at 36 volt value of SV and Ton value is high, both these conditions, initiate violent sparks with in region of tool and work that leads to quick removal of metal and hence faster rate of material removal is obtained [9].

**Figure 8:**
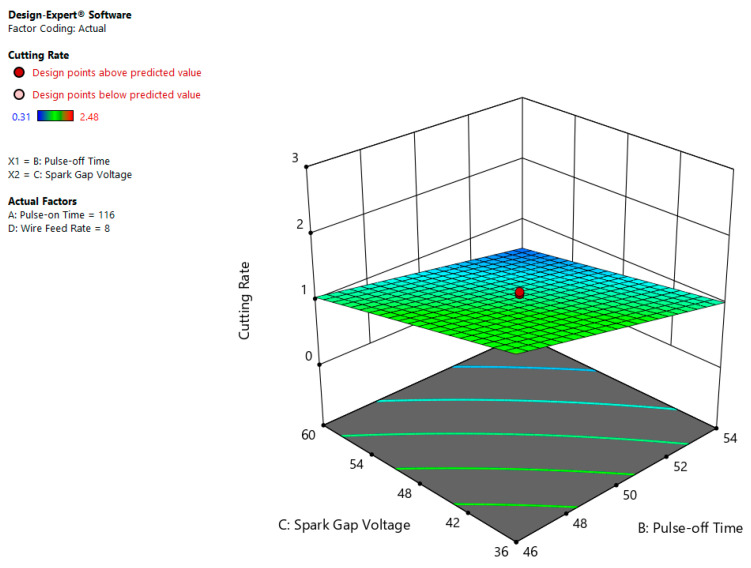
Correlational effect of Toff and SV on CR.

Figure 8 illustrates the impact of Toff and SV on CR, where low Toff (46 µs) value and low SV (36 volt) value indicated the higher CR (2.5 mm/min). This is due to the fact that as the servo voltage rises, the gap among work and tool increases, which leads to slow down the CR. However, reverse phenomenon has been observed as servo voltage decreases, then the CR increases due to less gap among tool and work leading to more melting and evaporation of work piece. So, it can be inferred from present case the CR is inversely proportional to the SV [16,28].

### 3.4. Statistical Observations for Surface Roughness (SR)

The F-value for SR as revealed in Table 6 is 24.24 which means that the obtained model is significant and tendency of error due to disturbance is only 0.01%. The ANOVA table suggested the importance of A, B, C, AB, AC, B^2^ and D^2^ entities. On the other hand, F-value in excess of 0.1000 means these model terms are not important. Furthermore, whenever there are numerous non-significant items then model reduction will enhance the problem. The lack of fit F-value of 2.59 means that the Lack of Fit in relation to the pure error is not important. Non-significant lack of fit is fine, because of the requirement to match the pattern.

The 0.7897 expected R^2^ is fairly in line with the 0.8651 Modified R^2^; i.e., difference is less than 0.2. Adeq-precision tests the ratio of a signal-to-noise. It is important to obtain a ratio greater than 4. This ratio of 16.798 indicates a suitable signal. The model may be used to traverse space in the design.

### 3.5. Regression Equation for SR

The equation for SR in terms of input variables is presented as under:

SR = 22.5835 − 0.27010 × Ton − 0. 03309 × Toff − 0.23687 × SV − 0.30125 × WF + 0.00515 × Ton × Toff + 0.00182 × Ton × SV − 0.00599 × Toff^2^ + 0.01945 × WF^2^

**Figure 9:**
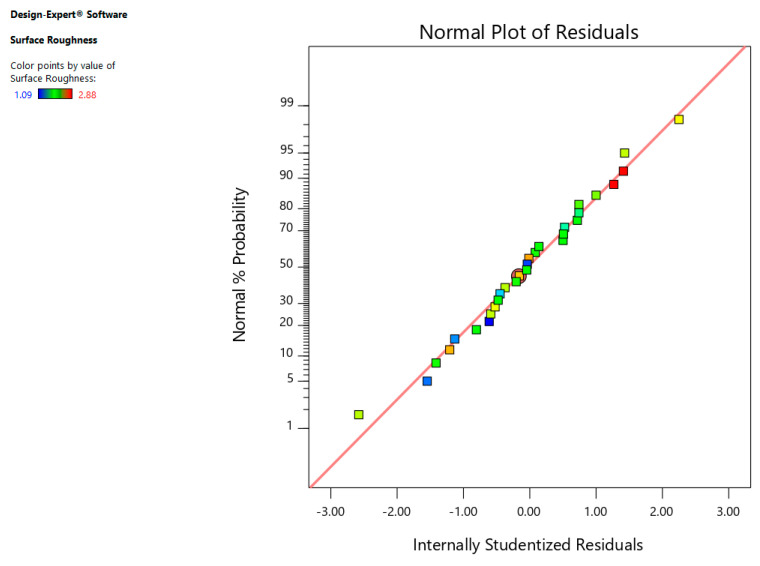
Normal chart of residual for surface roughness (SR).

**Figure 10:**
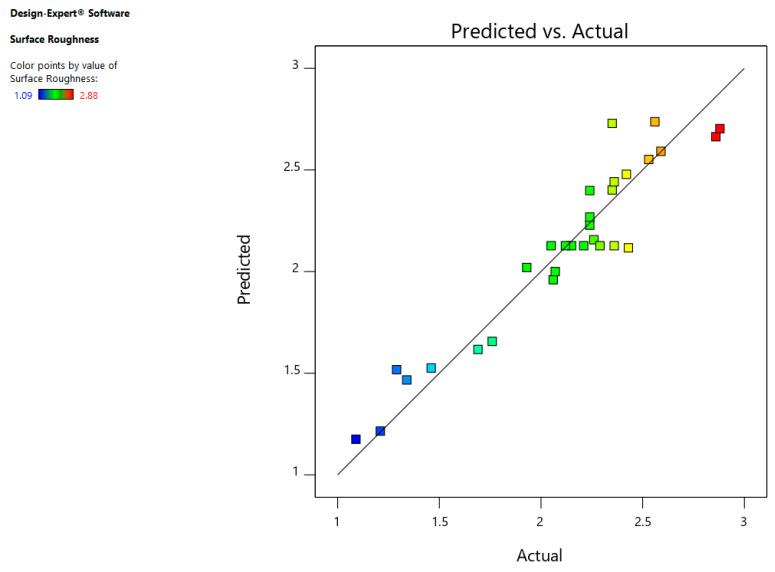
Predicted vs. actual plot for SR.

The residuals normal-probability plot indicates that data is aligned towards the straight line ensuring that data is normally distributed as shown in Figure 9. Furthermore, Figure 10 indicates that that whole experimental values and determined values from the equation are with in close range indicating the SR model is accurate.

### 3.6. Correlational Influence of Input Variable on SR

**Figure 11:**
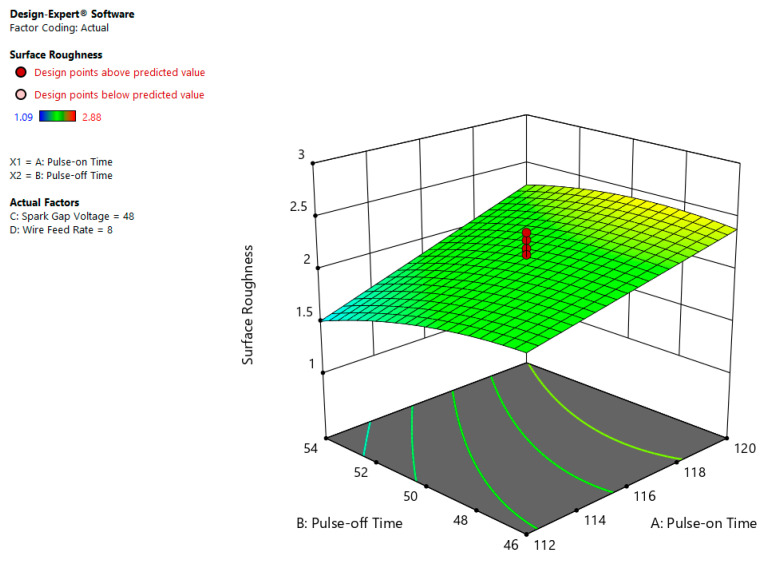
Correlation influence of Ton and Toff on SR.

The interaction effect is defined as the relation of process variable on outcome variables. Figure 11 indicates the impact of Ton and Toff on SR. From the plot it is visible that at higher Ton and lower Toff values, surface roughness is high because of more melting and evaporation of work material [9,28].

**Figure 12:**
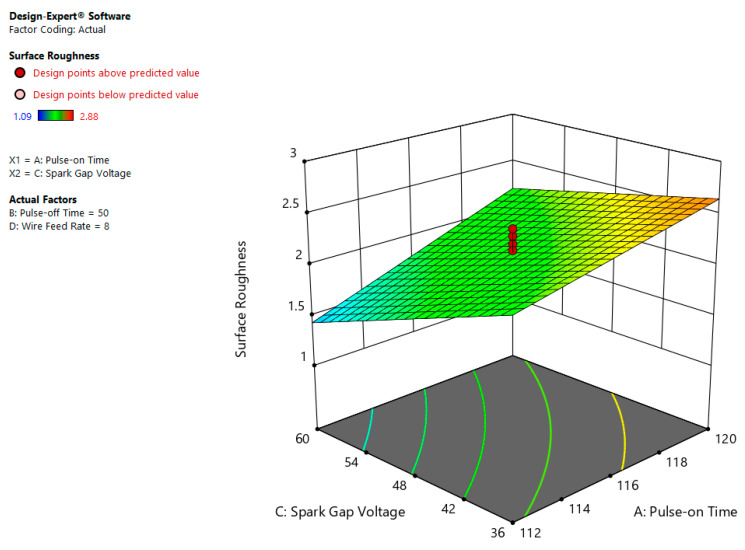
Interaction influence of Ton and SV on SR.

The correlation plot between Ton and SV is shown in Figure 12 which indicates that the SR is maximum at high value of Ton (120 µs) and least magnitude of SV (36 volt). From the plot, it is inferred that higher Ton and lower SV values lead to rise in discharge energy among work piece and tool. The more the discharge energy, the more the melting and evaporation of work material, thus as a result, there is increase in SR. Unlike, less magnitude of Ton and more value of SV reduces the discharge energy between job and electrode, therefore, leading to a fall in SR [28].

## 4. Multi-Response Optimization Using Desirability Function

Whenever there are large numbers of variables in a research problem and optimization of all of them is not possible during a single response output, then multi-objective optimization is the right solution. Therefore, in this work, multi-objective optimization has been executed by desirability criteria of RSM. The desirability function is applied to evaluate the optimum settings for WEDM process so as to find the best parameter range for maximizing CR and minimizing SR. As per the experimental results and analysis, the maximum and minimum limits of input and output parameters are shown in Table 7.

Table 8 displays the optimal values of WEDM variables that provides the high estimation of desirability for both single and multi-objective optimization. When performing single response optimization, the other response has been overlooked but both the responses were considered and given equal importance for multi-response optimization. The optimized CR and SR values obtained by multi-response optimization are 2.48 mm/min and 2.12 µm, respectively.

## 5. Conclusions

The selected input parameters (Ton, Toff, SV and WF) significantly affect the performance of the WEDM process. As per the experimental observations, the following conclusions have been obtained.

(a) The process parameters like Ton, Toff, SV have significant effect on for cutting-rate. The empirical relation is:
  CR = 11.23430 − 0.27541 × Ton + 0.070854 × Toff + 0.034292 × SV + 0.020625 × WF − 3.075× 10^−3^ ×Ton × Toff − 7.41667× 10^−4^ ×Ton × SV + 6.25000 × 10^−4^ × Toff × SV + 2.40938 × 10^−3^ × Ton^2^ + 1.85938× 10^−3^ × Toff^2^
(b) The process parameters like Ton and SV have major effect on for SR. The empirical relation is:
  SR = 10.51207 − 0.15017 × Ton + 0.06061 × Toff − 0.13619 × SV + 3.15000 × 10^−3^ × Ton × Toff + 1.01667 × 10^−3^ ×Ton × SV − 4.50693 × 10^−3^ × Toff^2^ + 5.47585 × 10^−4^ × WF^2^
(c) Analysis of response surfaces exhibited that Ton has influenced the cutting rate in such as a manner that during a rise in Ton, the cutting rate goes on increasing; however, it impacted surface-roughness catastrophically. Furthermore, it was noticed that the CR as well as SR both reduces as there is increment in pulse-off time.
(d) Moreover, it has been found that that CR decreases with rise in SV and vice versa. On the contrary, SR increases with decline in SV and vice versa. Also, the impact of wire-feed rate on the CR and SR has been found to be negligible.
(e) The optimization of a multi-response approach by giving equal priority to both the responses achieved the highest cutting rate of 2.48 mm/min, and the lowest roughness of 2.12 µm.

## Figures and Tables

**Table 1:** Chemical characteristics of Monel K-500.

Element	Ni	Cu	Fe	S	C	Mn	Si	Al	Ti
Weight (%)	65	27	2	0.01	0.25	1.5	0.5	2.30–3.15	0.35–0.85

**Table 2:** Parameters and levels selected through preliminary experiments.

S. No.	Parameters	Units	Range	Levels				
				1	2	3	4	5
Coded Values				−2	−1	0	1	2
1.	Pulse-on Time (A)	(µs)	108–124	108	112	116	120	124
2.	Pulse-off Time (B)		42–58	42	46	50	54	58
3.	Spark Gap Voltage (C)	(volts)	24–72	24	36	48	60	72
4.	Wire Feed rate (D)	(m/min)	4–12	4	6	8	10	12

**Table 3:** Fixed parameters and values.

S. No.	Fixed Parameter	Value
1	Peak-current	120 amperes
2	Peak-voltage	110 DC (maximum)
3	Wire tension	8 units
4	Servo feed	2100 units
5	Water pressure	15 kg/cm^2^

**Table 4:** Experimental observations.

Std Order	Run Order	“Pulse-on Time” (µs)	“Pulse-off Time” (µs)	“Servo-Voltage” (volts)	“Wire-Feed Rate” (m/min)	“Cutting-Rate” (mm/min)	“Surface-Roughness” (µm)
1	15	112	46	36	6	1.22	2.35
2	30	120	46	36	6	2.06	2.86
3	17	112	54	36	6	0.59	2.06
4	13	120	54	36	6	1.43	2.53
5	27	112	46	60	6	0.64	1.69
6	16	120	46	60	6	1.55	2.24
7	7	112	54	60	6	0.31	1.09
8	12	120	54	60	6	0.8	2.43
9	5	112	46	36	10	1.26	2.36
10	11	120	46	36	10	2.48	2.88
11	26	112	54	36	10	0.7	2.07
12	28	120	54	36	10	1.44	2.59
13	1	112	46	60	10	0.71	1.76
14	21	120	46	60	10	1.58	2.24
15	20	112	54	60	10	0.35	1.21
16	29	120	54	60	10	0.87	2.26
17	19	108	50	48	8	0.38	1.46
18	9	124	50	48	8	2.28	2.35
19	24	116	42	48	8	1.84	1.93
20	3	116	58	48	8	0.62	1.34
21	10	116	50	24	8	1.71	2.56
22	25	116	50	72	8	0.41	1.29
23	8	116	50	48	4	1.01	2.24
24	14	116	50	48	12	1.11	2.42
25	2	116	50	48	8	1.04	2.21
26	23	116	50	48	8	1.01	2.15
27	22	116	50	48	8	1.13	2.29
28	6	116	50	48	8	1.15	2.05
29	18	116	50	48	8	1.08	2.12
30	4	116	50	48	8	1.17	2.36

**Table 5:** Analysis of variance (ANOVA) test results for cutting rate (CR).

Source	“Sum of Squares”	“Degree of Freedom”	“Mean-Square”	“F-Value”	“Prob > F”	
Model	8.99	8	1.12	128.04	<0.0001	Significant
A-Pulse-on Time	4.36	1	4.36	496.87	<0.0001	
B-Pulse-off Time	2.31	1	2.31	263.52	<0.0001	
C-Spark Gap Voltage	2.02	1	2.02	230.65	<0.0001	
D-Wire Feed Rate	0.0408	1	0.0408	4.65	0.0427	
AB	0.0977	1	0.0977	11.13	0.0031	
AC	0.0452	1	0.0452	5.15	0.0340	
BC	0.0315	1	0.0315	3.59	0.0720	
A^2^	0.0767	1	0.0767	8.74	0.0075	
Residual	0.1843	21	0.0088			
Lack-of-fit	0.1640	16	0.0102	2.52	0.1562	Not-significant
Pure error	0.0203	5	0.0041			
Cor total	9.17	29				
Standard deviation	0.0937		R^2^		0.9799	
Mean	1.13		Adj. R^2^		0.9723	
CV%	8.28		Pred. R^2^		0.9503	
			Adeq. Precision		41.6418	

**Table 6:** ANOVA test results for SR.

Source	“Sum of Squares”	“Degree of Freedom”	“Mean-Square”	“F-Value”	“Prob > F”	
Model	5.59	8	0.6990	24.24	<0.0001	Significant
A-Pulse-on Time	2.17	1	2.17	75.33	<0.0001	
B-Pulse-off Time	0.4593	1	0.4593	15.93	0.0007	
C-Spark Gap- Voltage	2.23	1	2.23	77.43	<0.0001	
D-Wire Feed-Rate	0.0096	1	0.0096	0.3329	0.5701	
AB	0.1089	1	0.1089	3.78	0.0655	
AC	0.1225	1	0.1225	4.25	0.0519	
B^2^	0.2618	1	0.2618	9.08	0.0066	
D^2^	0.1722	1	0.1722	5.97	0.0234	
Residual	0.6055	21	0.0288			
Lack-of-fit	0.5404	16	0.0338	2.59	0.1487	Not-significant
Pure-error	0.0651	5	0.0130			
Cor-total	6.20	29				
Standard-deviation	0.1698		R^2^		0.9023	
Mean	2.11		Adj. R^2^		0.8651	
CV%	8.04		Pred. R^2^		0.7897	
			Adeq. Precision		16.7976	

**Table 7:** Constraints for input and output parameters.

Constraints	To Achieve	Limit (lower)	Limit (Upper)	Important
Ton (µs)	In range	108	124	3
Toff (µs)		42	58	3
SV (volts)		24	72	3
WF (m/min.)		4	12	3
CR (mm/min.)	Maximize	0.31	2.48	3
SR (µm)	Minimize	1.09	2.88	3

**Table 8:** Single and multi-objective optimization for desirability at high value.

Response	Process Parameters				Predicted Response		Desirability
	Ton (µs)	Toff (µs)	SV (volts)	WF (m/min)	CR (mm/min)	SR (µm)	
Single response optimization to maximize CR	121	45	25	6	2.91	-	1.000
Single response optimization to minimize SR	114	57	68	8	-	0.88	1.000
Multi response optimization to maximize CR and minimize SR	124	42	60	8	2.48	2.12	0.689

## Nomenclature

Adeq. Precision	Adequate Precision
Adj. R2	Adjusted-R^2^
ANOVA	Analysis of Variance
CCD	Central Composite Design
CNC	Computer Numerical Control
CR	Cutting Rate
CV	Coefficient of Variation
DOE	Design of Experiment
Pred. R^2^	Predicted R^2^
R^2^	Determination Coefficient
RSM	Response Surface Methodology
SV	Spark Gap Voltage
SR	Surface Roughness
Toff	Pulse-off Time
Ton	Pulse-on Time
WEDM	Wire Electrical Discharge Machining
WF	Wire-feed rate
